# Gut microbiota in hypothyroidism: pathogenic mechanisms and opportunities for precision microbiome interventions

**DOI:** 10.3389/fmicb.2025.1661211

**Published:** 2025-10-01

**Authors:** Tao Jiang, Xiuqing Yang, Baihui Wu, Runchao Tao, Rongbing Chen, Libo Jin, Da Sun, Huibin Weng

**Affiliations:** ^1^Institute of Life Sciences & Biomedical Collaborative Innovation Center of Zhejiang Province, Wenzhou University, Wenzhou, China; ^2^College of Life and Environmental Science, Wenzhou University, Wenzhou, China; ^3^Department of Biomedical Engineering, City University of Hong Kong, Hong Kong, Hong Kong SAR, China; ^4^The Quzhou Affiliated Hospital of Wenzhou Medical University, Quzhou People’s Hospital, Quzhou, China

**Keywords:** gut microbiota, hypothyroidism, immune regulation, probiotics, prebiotics, microbiota-targeted therapy

## Abstract

Hypothyroidism is a common endocrine disorder characterized by insufficient thyroid hormone synthesis or secretion, most frequently caused by Hashimoto’s thyroiditis, an autoimmune condition that leads to chronic thyroid gland damage. Despite the widespread use of levothyroxine replacement therapy, a substantial proportion of patients continue to experience persistent symptoms and metabolic dysregulation even after achieving biochemical euthyroidism. These observations have prompted growing interest in non-hormonal contributors to hypothyroidism, particularly the role of the gut microbiota. Recent studies indicate that gut microbial dysbiosis may influence the onset and progression of hypothyroidism through mechanisms involving immune dysregulation, increased intestinal permeability, chronic low-grade inflammation, and impaired nutrient absorption. Additionally, microbial metabolites such as short-chain fatty acids and bile acids are increasingly recognized as modulators of thyroid hormone metabolism, tissue sensitivity, and enterohepatic circulation. The bidirectional interactions between thyroid function and the gut microbiota constitute the emerging concept of the gut–thyroid axis, providing a novel framework for understanding the disease. Microbiota-targeted interventions, including probiotics, prebiotics, synbiotics, and dietary modulation, have demonstrated potential to improve microbial composition, alleviate systemic inflammation, enhance thyroid hormone utilization, and reduce autoantibody levels. This review systematically explores the mechanistic links between gut microbiota and hypothyroidism, critically evaluates current microbiota-based therapeutic strategies, and highlights future opportunities for personalized, microbiome-driven interventions to optimize the management of hypothyroidism.

## 1 Introduction

Hypothyroidism is a prevalent endocrine disorder defined by inadequate synthesis or secretion of thyroid hormones, primarily thyroxine (T 4) and triiodothyronine (T 3) (McDermott, 2020). These hormones are critical for regulating basal metabolic rate, thermogenesis, lipid and glucose metabolism, as well as neurocognitive development (Arrigo et al., 2008). A deficiency in thyroid hormones leads to a wide range of clinical manifestations, including fatigue, bradycardia, cold intolerance, weight gain, constipation, depression, and cognitive impairment (Taylor et al., 2024). Hashimoto’s thyroiditis, a chronic autoimmune condition characterized by lymphocytic infiltration of the thyroid gland and the presence of elevated antithyroid antibodies such as thyroid peroxidase antibodies (TPOAb) and thyroglobulin antibodies (TgAb), remains the primary cause of hypothyroidism in iodine-sufficient populations (Liu et al., 2022). Epidemiological studies estimate that the global prevalence of overt hypothyroidism is approximately 4.6% in the United States and 5% in Europe, with even higher rates observed in iodine-deficient regions (Persani, 2012). Although its pathogenesis may vary depending on geography, genetic factors, and environmental influences, hypothyroidism is often considered a chronic and ongoing health problem (Centanni et al., 2017; Stabouli et al., 2010).

Early diagnosis and timely treatment are essential to alleviate symptoms, prevent complications, and improve quality of life. The currently standard of care involves levothyroxine replacement therapy, which is typically effective in restoring serum hormone levels to the euthyroid range (Bianco and Taylor, 2024). However, a significant proportion of patients continue to experience persistent symptoms despite achieving biochemical normalization (Biondi and Cooper, 2019). These cases have led to growing interest in identifying non-hormonal contributors to disease burden, including immune system activation, micronutrient malabsorption, and alterations in the gut microbiota. Indeed, increasing evidence suggests that the gut microbiota plays an integral role in the pathophysiology of hypothyroidism (Samuels and Bernstein, 2022). Beyond its classical functions in digestion and nutrient absorption, the gut microbiota is involved in regulating immune homeostasis, modulating systemic inflammation, maintaining epithelial barrier integrity, and influencing host metabolism (Adak and Khan, 2019; Fröhlich and Wahl, 2019; Yan et al., 2024). Modern lifestyle factors such as poor dietary habits, psychological stress, polypharmacy, and exposure to environmental toxins frequently disrupt microbial balance and lead to dysbiosis, a state increasingly linked to numerous systemic conditions, including endocrine disorders (Ma et al., 2022; Mangiola et al., 2016; Emm, 2017; Qiu et al., 2022; Wang X. et al., 2024).

In the context of hypothyroidism, both clinical and experimental studies have reported alterations in gut microbial composition. These include reduced microbial diversity, decreased abundance of beneficial taxa such as *Bifidobacterium* and *Faecalibacterium prausnitzii*, and increased representation of pro-inflammatory microbes (Khavandegar et al., 2024; Weiss et al., 2010; Shi et al., 2024). These changes have been associated with increased intestinal permeability, elevated systemic endotoxin levels, and immune dysregulation, all of which may contribute to the development and progression of thyroid dysfunction (Miquel et al., 2013; Qu et al., 2023; de Vos et al., 2022). Moreover, microbial metabolites such as short-chain fatty acids and secondary bile acids influence thyroid hormone metabolism by regulating hepatic deiodinase activity, enterohepatic circulation, and tissue sensitivity to T 3 (Miquel et al., 2013; Su et al., 1979). At the same time, thyroid hormone deficiency may impair gastrointestinal motility and alter the mucosal environment, further disrupting microbial composition and reinforcing a bidirectional relationship between thyroid function and gut health.

**Figure 1:**
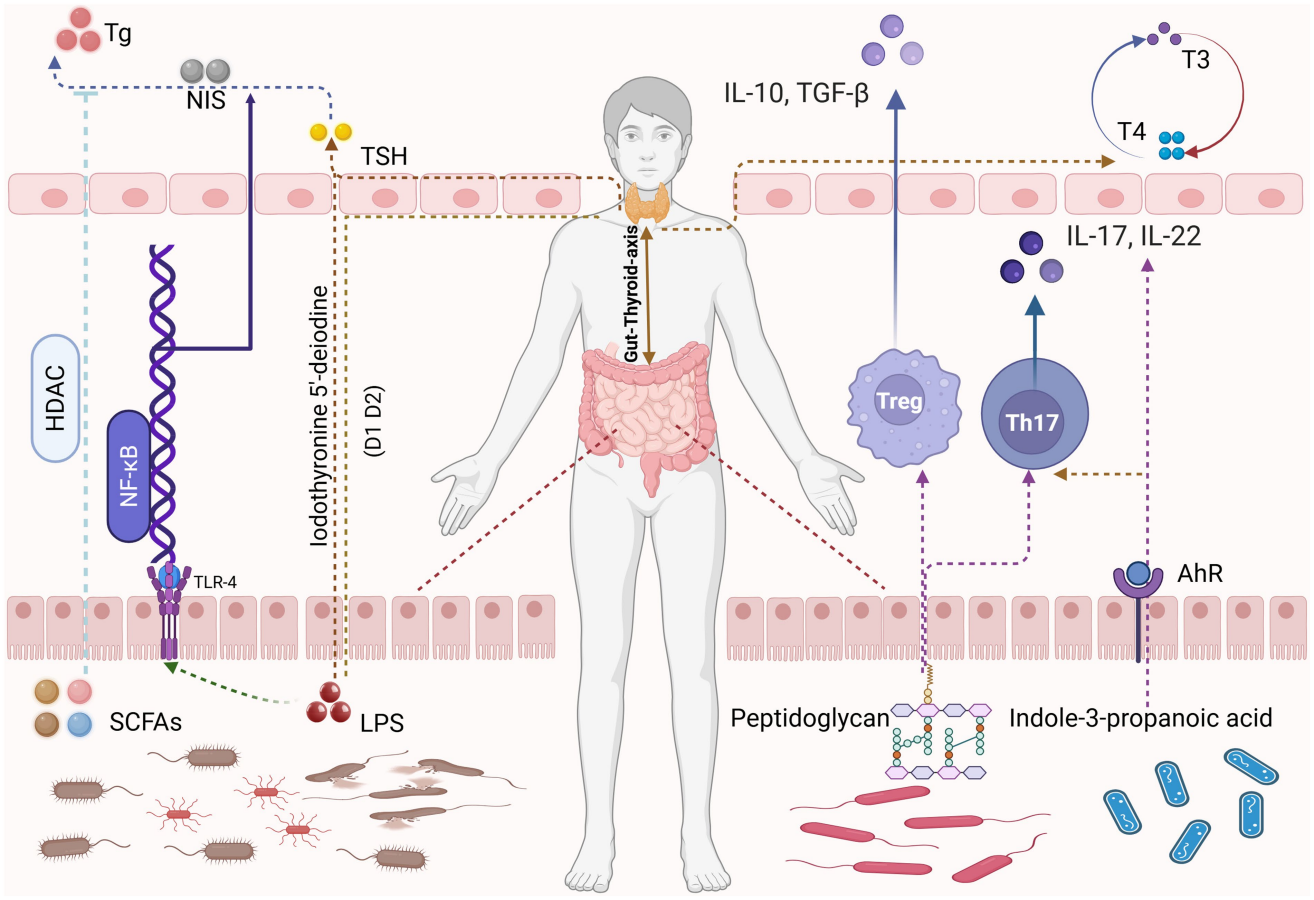
Certain gut microbiota metabolites, such as SCFAs and LPS, regulate thyroid hormone production and metabolism through HDAC signaling pathways and NF-*κ*B signaling pathways, respectively. Meanwhile, metabolites produced by certain probiotics including peptidoglycan and indole-3-propanolic acid—modulate the human immune system by influencing Tregs and Th17 cells, thereby maintaining thyroid homeostasis. Image source: used BioRender.com created.

As shown in Figure 1, the concept of a gut-thyroid axis provides a compelling framework for understanding these interactions (Virili et al., 2024; Su et al., 1979; Huang et al., 2025; Yu et al., 2021; Jiang et al., 2022). Given their shared embryological origins, gut and thyroid follicular cells exhibit structural and functional parallels, potentially facilitating reciprocal influences that impact disease pathogenesis and progression (Lahner et al., 2020). The common origin may be the basis for explaining the interaction between the two. When the gut microbiota changes, it may indirectly lead to abnormalities in thyroid function. For example, certain gut microbes may affect hormone levels in the thyroid gland by producing metabolites that affect hormone synthesis or metabolism (Liu X. et al., 2024). This has spurred interest in microbiota-targeted interventions, including probiotics, prebiotics, and dietary modulation. Low-quality evidence from two randomized controlled trials suggests that routine administration of probiotics, prebiotics, or synbiotics may provide minimal benefit for patients with primary hypothyroidism. While probiotic/prebiotic supplementation does not affect thyroid hormone levels, it may modestly reduce TRAb levels in Graves’ disease patients (Zawadzka et al., 2023; Shu et al., 2024). Preliminary studies have shown that specific probiotic strains may enhance thyroid hormone bioavailability, reduce antithyroid antibody titers, and alleviate common symptoms such as fatigue and mood disturbances (Xie et al., 2023; Liu et al., 2023). In addition to exploring the scientific basis for these interactions, we sort out the clinical implications of targeting the gut microbiota in hypothyroidism management. Probiotic intake does not directly alter thyroid function compensation, but it helps prevent serum hormone fluctuations and maintains thyroid hormone homeostasis. Results from a two-sample Mendelian randomization study demonstrate a causal relationship between *Akkermansia* and hypothyroidism, indicating that *Akkermansia* may inhibit the occurrence and progression of hypothyroidism (Shi et al., 2024; Spaggiari et al., 2017).

To elucidate these emerging insights, this review focuses specifically on hypothyroidism, rather than thyroid disorders in general, and synthesizes current knowledge on the mechanistic, clinical, and therapeutic implications of gut microbiota in this context. We explore the key microbial shifts associated with autoimmune hypothyroidism, the potential pathways by which microbiota modulate thyroid function, and the therapeutic promise of microbiota-based interventions. Additionally, we discuss existing challenges and propose future directions for integrating microbiome science into the personalized management of hypothyroidism.

## 2 Association of hypothyroidism and the gut microbiota

**Figure 2:**
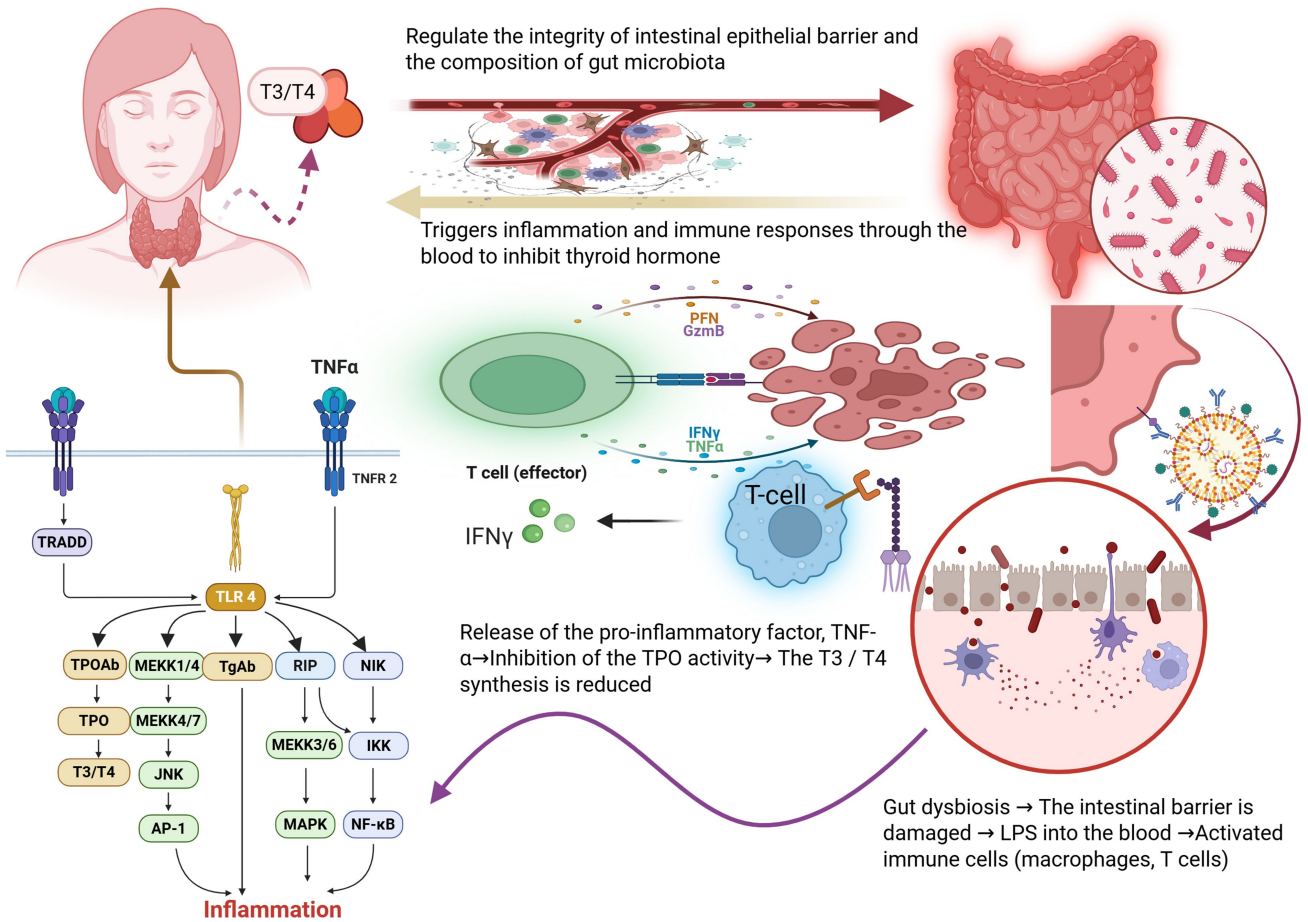
Disruption of gut microbiota directly damages the intestinal barrier, subsequently affecting the immune system. When TLR-4 receptors are activated, downstream signaling pathways such as MAPK and NF-κ B are triggered, which in turn stimulate the release of thyroid inflammatory factors TPOAb and TGAb, leading to hypothyroidism and reduced T3/T4 levels further compromise the stability of the intestinal barrier and disrupt the balance of gut microbiota. Image source: used BioRender.com created.

Recent advances in microbiome research have markedly expanded our understanding of how the gut microbiota critically influences human health, particularly through intricate interactions with endocrine disorders such as hypothyroidism. Central to these interactions is the gut-thyroid axis, a bidirectional communication pathway that has emerged as a pivotal factor in elucidating the pathogenesis and progression of thyroid diseases (Figure 2). Clinical and experimental studies increasingly demonstrate that shifts in gut microbial composition are significantly correlated with hypothyroidism, underscoring the complexity and importance of this relationship (Liu Y. et al., 2024; Nanda et al., 2008; Liu et al., 2023).

### 2.1 Changes in gut microbiota diversity associated with hypothyroidism

The diversity and stability of the gut microbiota are sensitive indicators of health status, and disruptions to this microbial ecosystem are strongly associated with various chronic conditions, including hypothyroidism. Analysis of the gut microbiota of the Hashimoto’s thyroiditis (HT) patient population and the healthy population revealed decreased richness and diversity of HT patients, especially those advancing to clinical hypothyroidism (Liu et al., 2020). Furthermore, *Lachnospiraceae, Lactonifactor*, *Alistipes* and *Subdoligranulum* were more abundant in HT patients with normal thyroid function while *Phascolarctobacterium* was more abundant in patients with hypothyroidism. Further analysis showed that *Phascolarctobacterium* is negatively correlated with multiple pathways including environmental information processing and metabolism (Liu et al., 2020). We speculate that *Phascolarctobacterium* may be involved in the development of HT. In the early development of HT, the *α* diversity of gut microbiota did not change significantly, but the flora structure had quietly changed. For example, Bacillota and Spirochaetota increased in abundance with significant divergence across multiple genera and species. Among them, produce acetate, butyrate of beneficial bacteria such as *Catonella*, *Murimonas intestini* and *Barnesiella intestinihominis* decreased, while harmful bacteria such as, *Klebsiella*, *Escherichia* and *Streptococcus* increased (Li et al., 2024). With the progression of HT, these imbalances intensify further, with a substantial decline in beneficial bacteria and heightened dominance of pathogenic bacteria, including *Streptococcus*, *Enterobacteriaceae* and *Acinetobacter* (Sessa et al., 2025). Although the number of species detected in the sample increased (increased species richness), some species may over proliferate, leading these species to dominate the community, thus reducing evenness (the Simpson index decreased). Consequently, patients with hypothyroidism may demonstrate diminished microbial diversity in their intestinal ecosystem, potentially leading to dysbiosis characterized by the disproportionate dominance of specific bacterial taxa.

### 2.2 Functional implications of specific gut microbiota in hypothyroidism

As seen in the results mentioned above, *Phascolarctobacterium* is more abundant in patients with hypothyroidism (Liu et al., 2020). *Phascolarctobacterium* participates in the negative regulation of most pathways, including cellular processes, environmental information processing, genetic information processing, and metabolism. *Phascolarctobacterium* can produce short-chain fatty acids (SCFAs), including acetate and propionate, as reported to be related to the metabolic state and mood of the host (Wu et al., 2017). In-depth analyses utilizing techniques such as Mendelian randomization have revealed specific changes in gut microbial communities associated with hypothyroidism. These studies report increased abundance of bacterial groups including *Negativicutes*, *Christensenellaceae*, *Selenomonadales*, and *Ruminococcus*, alongside a notable decrease in *Verrucomicrobia*, *Akkermansia muciniphila*, and *Erysipelotrichaceae* UCG003 showed an decrease (Liu et al., 2023). Patients with hypothyroidism have significant changes in the gut microbiota, and these changes are closely linked to the development of the disease, and have an important impact on the digestive, immune and metabolic functions of the body. In the normal physiological state, *Akkermansia muciniphila*, with its unique cellular structure and metabolites, is closely bound with intestinal epithelial cells to maintain the integrity of intestinal mucosa, prevent the invasion of harmful substances, and ensure the stability of the intestinal environment (Garcia-Vello et al., 2024). In hypothyroidism patients, *Akkermansia muciniphila* numbers are upregulated, which may be a compensatory response of the body facing the impaired intestinal barrier. On the one hand, *Akkermansia muciniphila* enhances the junction strength between intestinal epithelial cells by regulating the expression of tight junction proteins, such as ZO-1, Occludin, and Claudin-1, to attempt to repair the disrupted intestinal barrier (Liu et al., 2023). On the other hand, *Akkermansia muciniphila* can activate immune cells in the intestine, such as macrophages and dendritic cells, to secrete the anti-inflammatory cytokine IL-10 and inhibit the inflammatory response (Mei et al., 2024). However, when *Akkermansia muciniphila* proliferates excessively, it breaks the intestinal immune homeostasis. The structure of lipopolysaccharide (LPS) on the surface of *Akkermansia muciniphila* is different from other Gram-negative bacteria, which has immunomodulatory effect at low concentrations. But it will be recognized by the immune system in excess, activating the Toll-like receptor 4 (TLR 4) signaling pathway, and promoting the release of immune cells to release a large number of pro-inflammatory cytokines, such as TNF- *α* and IL-6 (Qu et al., 2023). These pro-inflammatory factors enter the blood circulation and trigger systemic inflammation that interferes with the synthesis and secretion of thyroid hormones. At the same time, the hyperactivated immune system mistakenly attacks thyroid tissue, producing thyroid autoantibodies, such as TPOAb and TgAb, which further damage thyroid function.

Conversely, the proliferation of harmful microbial taxa such as *Alistipes*, *Erysipelotrichaceae* UCG003, and *Gammaproteobacteria* adversely impacts thyroid health. *Alistipes* alters bile acid metabolism and affect the absorption of fat and fat-soluble vitamins, which participate in the synthesis and metabolism of thyroid hormones, thus indirectly affecting thyroid function (Parker et al., 2020). *Erysipelotrichaceae* UCG003 stimulates immune cells to release proinflammatory factors, triggering inflammation, which interfere with the activity of thyroid hormone synthetase (Huang et al., 2024). Additionally, *Gammaproteobacteria,* recognized as opportunistic pathogens, increases susceptibility to intestinal infections and exacerbate inflammation-driven metabolic dysfunction, impairing thyroid hormone utilization (Yao et al., 2020). The crucial gut microbiota and their mechanisms are systematically summarized in Table 1.

**Table 1:** Changes of gut microbiota in patients with hypothyroidism.

Classification	Strain	Species abundance	Mechanism	References
Ruminococcaceae	*Phascolarctobacterium*	Up-regulation	Participates in the negative regulation of most pathways and produce short-chain fatty acids	Liu et al. (2020) and Wu et al. (2017)
Spirochaetaceae	*Catonella*	Reduction	Produces butyrate and acetate	Li et al. (2024) and Zhao et al. (2017)
Barnesiellaceae	*Barnesiella_intestinihominis*	Reduction	Associated with LPS biosynthesis and SCFAs degradation, and regulating the immune system	Yan et al. (2022) and Doi et al. (2024)
Moraxellaceae	*Acinetobacter*	Reduction	Affects the local inflammatory response of the body	Chebotar et al. (2014)
Oscillospiraceae	*Ruminococcus*	Up-regulation	Reduces the intestinal epithelial energy supply deficit with impaired barrier function.	Hall et al. (2017)
Akkermansiaceae	*Akkermansia muciniphila*	Reduction	Promotes Treg cell differentiation and inhibit the Th 17 inflammatory response.	Cani et al. (2022), Rodrigues et al. (2022), and Macchione et al. (2019)
Erysipelotrichaceae	*Erysipelotrichaceae* UCG003	Reduction	Influences on the inflammatory response through the inflammatory proteins	Huang et al. (2024) and Shucheng et al. (2024)
Rikenellaceae	*Alistipes*	Up-regulation	Affects the body’s inflammatory response and oxidative stress	Parker et al. (2020) and David et al. (2014)

### 2.3 Mechanisms linking gut microbiota dysbiosis to hypothyroidism development

Gut microbiota dysbiosis exerts significant impacts on multiple physiological systems, directly and indirectly exacerbating the pathogenesis of hypothyroidism through several interconnected mechanisms. Primarily, hypothyroidism itself adversely affects gastrointestinal physiology by diminishing gastric mucosal integrity and reducing gastric acid secretion, thereby impairing the efficient absorption of nutrients and trace elements critical for thyroid function, such as selenium, iodine, iron, zinc, and vitamin D (Shulhai et al., 2024). Furthermore, hypothyroid-induced decreases in gastrointestinal motility often clinically present as bloating, constipation, and impaired digestion, which further disrupts microbial stability (Xu et al., 2024). Concurrently, the imbalance characterized by reduced beneficial bacteria such as *Bifidobacterium* and *Lactobacillus*, and proliferation of opportunistic pathogenic bacteria such as *Enterobacteriaceae* and *Streptococcus* significantly disrupts gut immune homeostasis, promoting chronic low-grade inflammation (Liu Y. et al., 2024). Dysbiosis-induced increased intestinal permeability allows the translocation of bacterial endotoxins, notably LPS, into the systemic circulation, triggering inflammatory cascades that compromise thyroid tissue integrity and hormone synthesis. Moreover, the imbalance in microbiota composition skews immune cell differentiation toward pro-inflammatory phenotypes, particularly Th 1 and Th 17 cells, and concurrently diminishes the function and numbers of regulatory T cells, thereby enhancing the risk and severity of autoimmune thyroid diseases (Takiishi et al., 2017).

Crucially, specific gut bacteria directly influence thyroid hormone metabolism through intrinsic enzymatic activities, such as bacterial deiodinase-like enzymes. These enzymes are capable of altering the critical peripheral conversion of inactive T 4 into the bioactive hormone T 3, profoundly influencing systemic thyroid hormone availability and function (Hoermann et al., 2016). Gut dysbiosis may also directly affect the level of thyroid hormone through its characteristic deiodinase activity. For example, some gut microbes have the ability to remove iodine atoms in hormones, which may lead to the inhibition of the synthesis and secretion of thyroid hormones (Köhrle et al., 1987). Furthermore, gut microbiota modulates the bioavailability, absorption, and metabolism of essential micronutrients required for thyroid hormone biosynthesis. Gut dysbiosis may thus result in deficiencies of critical micronutrients, notably iodine, selenium, iron, zinc, and vitamin D, which are indispensable for optimal thyroid function and regulation (Knezevic et al., 2020). In addition to these metabolic and nutritional pathways, gut microbiota dysbiosis also influences neuroendocrine regulatory mechanisms through modulation of neurotransmitter synthesis. Specifically, altered microbiota composition can disrupt dopamine metabolism and subsequently interfere with thyroid-stimulating hormone (TSH) secretion via the hypothalamic–pituitary-thyroid axis, further complicating thyroid homeostasis (Xie et al., 2023). Collectively, these multifaceted interactions emphasize the critical roles played by gut microbiota in the etiology and progression of hypothyroidism, underscoring the therapeutic potential of targeted microbiota interventions, including probiotics, prebiotics, dietary adjustments, and lifestyle modifications, to restore microbiome equilibrium and mitigate hypothyroid pathology.

## 3 Network analysis of host immune-inflammatory pathways and gut microbiota perturbations

**Figure 3:**
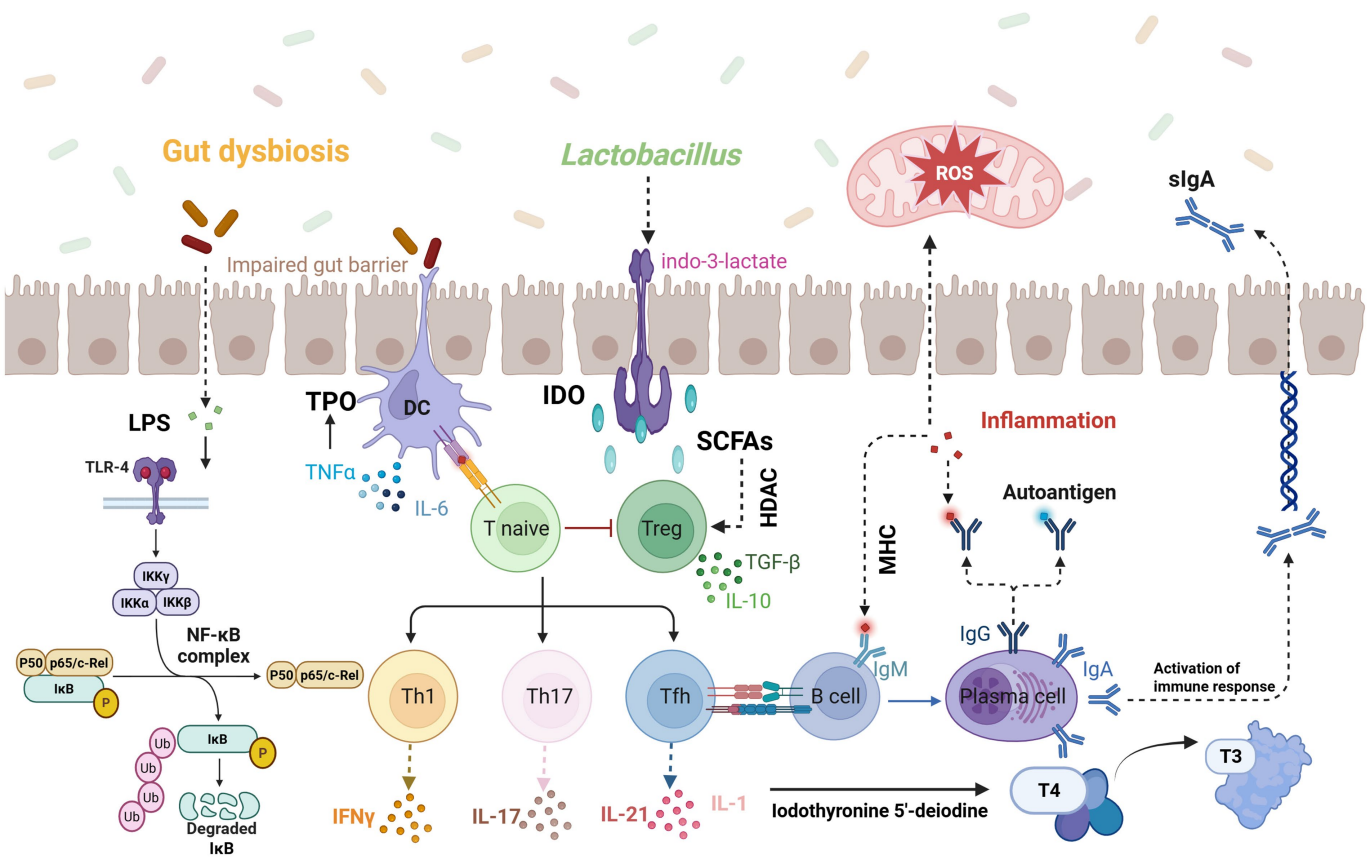
Disruption of gut microbiota compromises the intestinal barrier, allowing increased LPS exposure into the bloodstream which triggers TLR-4/NF-κB-mediated immune responses and inflammatory cytokine production; meanwhile, probiotics like Lactobacillus produce indole-3-lactate to activate dendritic cells, and SCFAs influence immune cell activation via HDAC signaling, ultimately leading to oxidative stress and IL-1-induced impairment of deiodinase synthesis, thereby disrupting thyroid hormone conversion (T3/T4). Image source: used BioRender.com created.

The intricate interplay between the gut microbiota and the host’s immune-inflammatory pathways is pivotal in maintaining endocrine homeostasis, particularly concerning thyroid function. Disruptions in this delicate balance can precipitate thyroid dysfunctions, notably hypothyroidism. This section delves into the mechanisms by which gut microbiota perturbations influence host inflammatory responses and immune regulation, contributing to the pathogenesis of hypothyroidism (Figure 3).

### 3.1 Inflammatory pathways mediated by gut dysbiosis

Gut dysbiosis, characterized by an imbalance in microbial composition, compromises intestinal barrier integrity, leading to increased permeability. This heightened permeability facilitates the translocation of bacterial endotoxins, such as LPS, into the systemic circulation. LPS interacts with TLR 4 on immune cells, activating the nuclear factor kappa-light-chain-enhancer of activated B (NF-*κ* B) cells signaling pathway (Peña-Durán et al., 2025). The activated NF-κ B rapidly translocated from the cytoplasm into the nucleus, binds to specific DNA sequences, induces the expression of several proinflammatory cytokine genes, and promotes the massive release of proinflammatory cytokines such as TNF-*α*, IL-1β, and IL-6 (Xu et al., 2022). These pro-inflammatory cytokines circulate in the blood, and have many multifaceted adverse effects on thyroid tissue. At the level of thyroid hormone synthesis, TNF-α can directly inhibit the activity of thyroid peroxidase (TPO), and IL-1 stimulates iodothyronine 5′-deiodine activity in the liver to affect the metabolism of thyroid hormone (Fujii et al., 1989). TPO is a key enzyme in the synthesis of thyroid hormone responsible for catalytic oxidation of iodine and iodization of tyrosine, and inhibition of TPO activity can directly lead to reduced thyroid hormone synthesis (Godlewska and Banga, 2019). Proinflammatory cytokines alter the expression of immune-related molecules on the surface of thyroid cells, such as the upregulation of major histocompatibility complex (MHC) molecules, making thyroid cells more likely to be recognized by the immune system and attacked by immune cells (Yan et al., 2024).

In addition, a persistent inflammatory state triggers a sharp increase in oxidative stress levels in thyroid tissue and the production of excessive reactive oxygen species (ROS). ROS attack lipids, proteins and DNA in thyroid cells. Specifically, ROS will cause lipid peroxidation of the cell membrane of thyroid cells, destroy the integrity and fluidity of the cell membrane, affect the uptake of iodine and other nutrients, and interfere with the synthesis of thyroid hormone (Ates et al., 2015). Meanwhile, ROS attack intracellular proteins, resulting in the abnormal function of many proteins involved in the synthesis, transport and regulation of thyroid hormones. Attack on DNA may trigger cell apoptosis or gene mutation, affect the normal proliferation and differentiation of thyroid cells, further damage the synthesis and secretion of thyroid hormone, and eventually aggravate the hypothyroidism condition (Lushchak, 2014).

### 3.2 Immune regulation disrupted by microbial imbalance

The gut microbiota plays a pivotal role in maintaining immune homeostasis, and its imbalance can significantly impact immune function. This means that substances, such as antigens, that are supposed to be restricted to the gut, can get into the circulation more easily. Because to the high exposure of antigen, the immune system overreacts. In the pathogenesis of hypothyroidism, especially autoimmune subtypes such as Hashimoto’s thyroiditis, gut microbiota plays a key role in the regulation of body immune homeostasis, affecting the occurrence and development of hypothyroidism through a series of complex mechanisms (Virili et al., 2018). Clinical studies have shown that the structure of the gut microbiota is significantly altered in hypothyroidism patients. The abundance of *Faecalibacterium*, a key genus responsible for SCFAs production, was significantly reduced, potentially leading to diminished butyrate synthesis and impaired gut homeostasis, while the abundance of proinflammatory bacteria *Prevotella* increased significantly (Li et al., 2024). SCFAs, especially butyrate, play a central role in immune regulation. Butyrate suppresses histone deacetylase (HDAC) and regulates Foxp 3 expression, which not only promotes the differentiation of Treg cells, but also enhances its inhibitory function (Mann et al., 2024). Meanwhile, SCFAs also suppresses STAT 3 phosphorylation and effectively reduces IL-6/IL-21-mediated Th 17 polarization (Anvar et al., 2024; Liu X. et al., 2024). This disrupts the balance between Th 17 and Treg cells, and the resulting proinflammatory microenvironment, dominated by Th 1 and Th 17 cells, further exacerbates the damage to thyroid tissue.

Changes in the gut microbiota can also damage the integrity of the intestinal barrier, which is an important factor in triggering systemic immune activation. Patients with hypothyroidism have increased intestinal permeability and “leaky gut” phenomenon, which makes the metabolic products of bacteria such as LPS and bacterial fragments easily translocate into the circulatory system, thus activating innate immunity (Tomov et al., 2005). LPS activates macrophages through TLR 4, prompting the release of inflammatory factors such as IL-1 *β* and TNF- *α*, which drive Th 1 differentiation and promote IFN- *γ* secretion, further magnifying local inflammation in the thyroid gland (Zhang et al., 2023). Additionally, IL-6 and IL-23 can drive Th 17 differentiation, with IL-17 recruiting neutrophils that infiltrate thyroid tissue, causing direct damage to follicular epithelial cells (Stadhouders et al., 2018). The gut microbiota’s influence extends to the modulation of autoimmune responses through molecular mimicry. Antigenic epitopes of specific gut microbiota such as *Bacteroides* have molecular similarities with thyroid peroxidase and thyroglobulin (Zafar and Saier, 2021). Certain bacterial antigens share structural similarities with thyroid autoantigens, such as TPO and thyroglobulin, leading to the production of cross-reactive autoantibodies like TPOAb and TgAb (Xu et al., 2021). This molecular mimicry can initiate and perpetuate autoimmune attacks on thyroid tissue, contributing to the progression of HT (Crotty, 2014). Furthermore, B cells can further differentiate into plasma cells and continuously produce autoantibodies (Vinuesa et al., 2016), which can mediate the complement-dependent thyroid cell killing process and further aggravate thyroid injury.

It is worth noting that the tryptophan metabolism is also disturbed in the intestine of hypothyroidism patients. Among them, tryptophan metabolic bacteria such as *Lactobacillus* decreased, resulting in a decrease in the activity of diamine 2,3-dioxygenase (IDO) and tryptophan metabolites such as indo-3-lactate can inhibit Th 17 differentiation and promote Treg function via the aromatic hydrocarbon receptor (Zelante et al., 2013; Rothhammer et al., 2016). At the same time, reduced IDO activity reduced kynurenine and further weakened peripheral immune tolerance, which played a driving role in the hypothyroidism immune disorder caused by the imbalance of the whole gut microbiota (Clarke et al., 2017).

### 3.3 Essential micronutrients and thyroid function

The intricate interplay between gut microbiota and the bioavailability of essential trace elements is pivotal for maintaining optimal thyroid function. Disruptions in the gut microbiome can adversely affect the absorption and metabolism of critical micronutrients, including iodine and selenium, thereby influencing thyroid hormone synthesis and immune regulation. Iodine is a fundamental component in the synthesis of thyroid hormones, and its deficiency is a well-established cause of hypothyroidism (McDermott, 2020). Emerging evidence suggests that gut microbiota can modulate iodine uptake and metabolism. Alterations in the gut microbiome may influence the expression and activity of the sodium/iodine symporter (NIS), a protein essential for iodine transport into thyroid follicular cells (Nicola et al., 2009). Metabolites produced by gut bacteria, such as LPS, have been implicated in affecting NIS functionality, thereby impacting iodine availability for thyroid hormone production.

Selenium is another trace element integral to thyroid health, predominantly due to its incorporation into selenoproteins (Ventura et al., 2017). These selenoproteins, including glutathione peroxidases and thioredoxin reductases, serve critical antioxidant functions, protecting thyroid tissue from oxidative damage during hormone synthesis (Schomburg, 2012; Mao et al., 2016). Additionally, iodothyronine deiodinases, which are selenium-dependent enzymes, regulate the conversion of T 4 to the biologically active T 3 (Arthur et al., 1992). Selenium deficiency can lead to decreased deiodinase activity, resulting in reduced T 3 levels and accumulation of inactive metabolites, thereby disrupting metabolic processes. Furthermore, inadequate selenium impairs the antioxidant defense system within the thyroid, increasing susceptibility to oxidative stress and inflammation (Avery and Hoffmann, 2018; Huang et al., 2012; Winther et al., 2020). Selenium supplementation improves the function of the thyroid and immune systems, thus correcting the interaction between lymphocytes and thyroid autoantigens in selenium-deficient patients. Restoring gut microbiota balance may enhance selenium absorption and utilization, thereby supporting thyroid hormone synthesis and mitigating inflammatory responses.

Vitamin D, while not a trace element, plays a significant role in immune modulation and has been linked to thyroid health. Studies have demonstrated an association between low vitamin D levels and autoimmune thyroid diseases, such as HT (Appunni et al., 2021). Vitamin D deficiency affects the absorption of calcium in the intestine, which may reduce the concentration of calcium ions in the body, affect the transmission of TSH signal, and stimulate the compensatory secretion of more TSH in the pituitary gland (Babić Leko et al., 2023). Moreover, vitamin D deficiency has been identified as a potential risk factor for the development of hypothyroidism and thyroid autoimmunity (Tang et al., 2023; Chahardoli et al., 2019). Supplementation with vitamin D has been observed to reduce TSH levels and decrease the prevalence of hypothyroidism, suggesting its role in modulating immune responses related to thyroid function (Safari et al., 2023; Pleić et al., 2024). Studies have also found that vitamin D supplementation can significantly reduce TPOAb levels (Chaudhary et al., 2016), suggesting that vitamin D may affect thyroid function via immunomodulation.

## 4 Probiotics and prebiotics as adjunctive therapeutic strategies in hypothyroidism

**Figure 4:**
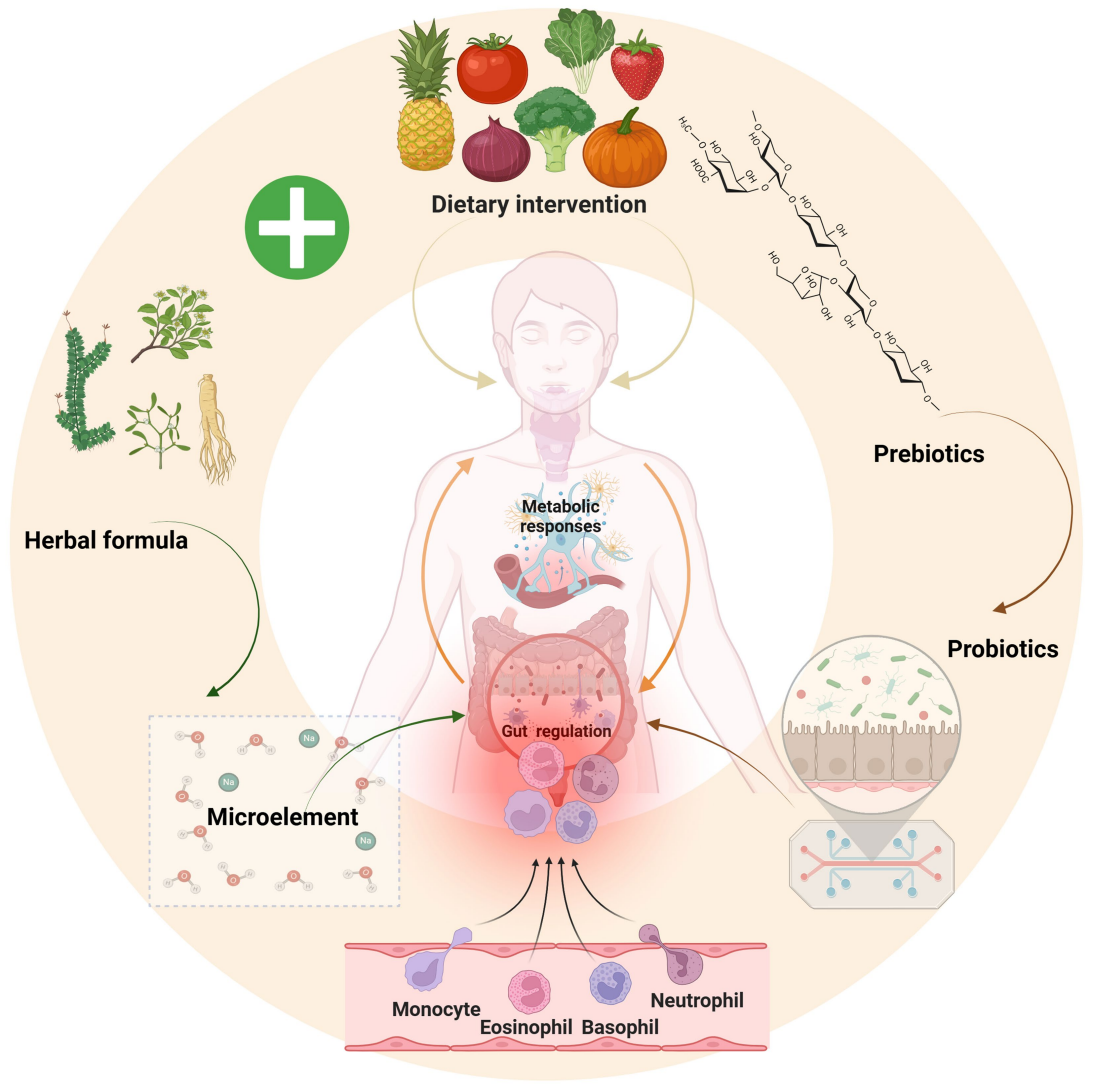
Polysaccharides from vegetable and fruit breakdown act as prebiotics to promote probiotic growth, which supply essential trace elements and regulate immune function; notably, traditional formulations like Yijung-tang exhibit similar prebiotic effects, suggesting that combining a green diet with probiotic-prebiotic therapy offers a novel strategy for hypothyroidism treatment. Image source: used BioRender.com created.

The recognition of the gut-thyroid axis as a pivotal regulator of endocrine and immune homeostasis has spurred increasing interest in microbiota-targeted interventions for hypothyroidism. Probiotics and prebiotics have demonstrated considerable potential as adjunctive strategies by reshaping the gut microbiome, modulating immune responses, optimizing metabolic function, and mitigating oxidative stress (Figure 4). These effects are particularly significant given the complex interplay between hypothyroidism, immune dysregulation, and metabolic disturbances (Fujii et al., 1989; Lushchak, 2014; Xu et al., 2021). In the field of hypothyroidism treatment, the mechanism of action and clinical potential of probiotics as adjuvant drugs have attracted much attention. Probiotics have a positive and profound impact on body metabolism, drug metabolism, thyroid function and systemic symptoms through multidimensional regulation mechanisms (Shulhai et al., 2024; Xie et al., 2023). At the same time, prebiotics, as indigestible food components, further enhance the role of probiotics by selectively stimulating the growth of specific beneficial bacteria in the gut (Sanders et al., 2019; Ouyang et al., 2024).

### 4.1 Therapeutic potential of probiotics in hypothyroidism: mechanistic insights

The gut microbiota plays a key role in energy metabolism, and probiotics can optimize the structure of the gut microbiota and reshape the microecological balance (de Vos et al., 2022). Probiotics, defined as live microorganisms that confer health benefits when administered in appropriate amounts, exert systemic regulatory effects highly relevant to the management of hypothyroidism. For example, *Bifidobacterium* promote the proliferation of beneficial commensals and inhibit the growth of pathogenic bacteria, thereby improving intestinal barrier function and nutrient absorption efficiency (Li et al., 2025). Efficient uptake of iodine, selenium, iron, and zinc—micronutrients governing thyrocyte function from hormone synthesis (iodination, TPO activity) to peripheral activation (DIO-mediated T 3 generation)—serves as a therapeutic target in hypothyroidism and Hashimoto’s thyroiditis, particularly in populations with coexisting deficiencies (Shulhai et al., 2024). Some probiotics, such as *Lactobacillus acidophilus*, can sense the changes in the intestinal environment, regulate the function of intestinal endocrine cells, and promote the secretion of gastrointestinal hormones such as glucagon-like peptide-1 (GLP-1) (Smith et al., 2019). GLP-1 can stimulate insulin secretion from islet *β* cells, enhance insulin sensitivity and maintain dynamic balance of blood glucose. Stable blood glucose level is crucial to the function of thyroid hormone to regulate glucose metabolism, and the precise regulation of blood glucose by probiotics provides a guarantee for the stability of thyroid function (Zhang et al., 2024b).

In terms of lipid metabolism, *Lactobacillus rhamnosus* can inhibit the intestinal absorption of cholesterol, promote the conversion of cholesterol and excretion of bile acid, and reduce the level of blood cholesterol (Zaccaria et al., 2023). At the same time, *Lactobacillus rhamnosus* derived extracellular Vesicles can regulate the process of adipocyte metabolism, inhibit the excessive release of inflammatory factors, reduce the chronic inflammatory reaction of adipose tissue, and improve dyslipidemia (Tong et al., 2021). In view of the mutual influence between dyslipidemia and hypothyroidism, the optimal regulation of blood lipid by probiotics can indirectly promote the recovery of thyroid function and form a virtuous cycle (Nanda et al., 2008; Ozair et al., 2018). In exploring the pathogenesis of autoimmune thyroiditis, it has been found that increased intestinal permeability enables toxins, antigens, or bacterial metabolites to enter the blood from the gut and lead to disease (Shi et al., 2024; Su et al., 1979). Probiotics as *Lactocaseibacillus rhamnosus* zz-1 can enhance tight junction protein expression (including ZO-1, occludin, and claudin-1) and promote epithelial barrier repair, thus limiting antigenic load and reducing systemic inflammation (Xu et al., 2023).

In addition, probiotics can also occupy the intestinal mucosal surface through competitive exclusion mechanism and reduce the colonization opportunity of pathogens. When the intestinal barrier is damaged, resulting in congenital immune dysregulation, the adaptive immune system becomes crucial. SCFAs, including butyrate, play a crucial role in regulating the balance between Th 17 and regulatory T cells (Yoo et al., 2020). This metabolic-immune crosstalk establishes mucosal tolerance while curbing pathological inflammation (Smith et al., 2013). At the same time, probiotics can also promote the secretion of digestive fluids (gastric juice, bile and pancreatic juice), enhance the activity of digestive enzymes such as amylase and lipase, optimize the process of food decomposition and absorption, and indirectly relieve the digestive dysfunction of hypothyroid patients (Lu et al., 2019). Therefore, the restoration of gut health and immune regulatory functions by probiotics may provide novel insights for the prevention and treatment of hypothyroidism.

### 4.2 Prebiotics as critical modulators of gut-thyroid homeostasis

Prebiotics, indigestible dietary fibers that selectively stimulate the growth and activity of beneficial gut bacteria, serve as essential adjuncts to probiotic therapy and independently contribute to hypothyroidism management (Ouyang et al., 2024). Prebiotics, as the “food” of probiotics, play an important role in optimizing the intestinal microecology and promoting the growth and function of probiotics. For example, prebiotics such as fructose oligosaccharides and galactose are able to significantly promote the proliferation of *Bifidobacteria* and *Lactobacillus*, while inhibiting the growth of harmful bacteria such as *Clostridium* and *Escherichia coli* (Wang K. et al., 2024). This effect not only optimizes the intestinal microecology, but also further improves the metabolic state of the body by enhancing the function of probiotics (Hijová et al., 2019).

The results showed that probiotic/prebiotic supplementation had no significant direct effect on TSH, free thyroxine and free triiodothyronine levels but significantly reduced thyrotrophin receptor antibody levels, revealing the potential value of probiotics and prebiotics in regulating autoimmune responses (Sanders et al., 2019). A limited number of studies have shown that probiotic products have certain effects on levothyroxine metabolism and thyroid hormone activity. The study found that after 8 weeks of intervention with synbiotic group, the concentration of thyroid-stimulating hormone, levothyroxine dosage and severity score of fatigue were significantly reduced (Talebi et al., 2020). The combinatorial application of prebiotics and probiotics can further improve the intestinal microecological environment through synergistic effects. For example, *β*-glucan can serve as a specific nutrient substrate for *Lactobacillus plantarum*, promoting its proliferation and activity. *Lactobacillus plantarum* in turn produces beneficial metabolites, strengthens gut epithelial defenses, enhance immune tolerance, and contributes to the restoration of thyroid immune homeostasis (Talebi et al., 2020; Siezen and van Hylckama Vlieg, 2011).

### 4.3 Emerging microbiota-targeted strategies for the management of hypothyroidism

Advances in microbiome research have unveiled novel strategies that complement traditional therapies for hypothyroidism. These emerging approaches focus on restoring gut homeostasis, modulating immune responses, and protecting thyroid function, addressing the multifaceted pathophysiology that underpins hypothyroid disorders. Drawing upon a comprehensive understanding of the aforementioned mechanisms and adopting a translational medicine approach, intervention strategies that target the gut microbiota possess substantial clinical significance. Specifically, the supplementation of *Faecalibacterium prausnitzii* or butyrate precursors (such as resistant starch) can effectively restore the levels of SCFAs, reinforce gut epithelial integrity, and reduce systemic inflammation (Miquel et al., 2013; Effendi et al., 2022). In parallel, traditional herbal formulations like Yijung-tang exert prebiotic-like effects, selectively enriching beneficial microbial taxa and promoting brown adipose tissue thermogenesis (Khakisahneh et al., 2023).

Probiotic intervention demonstrates significant therapeutic efficacy in treating autoimmune-mediated hypothyroidism caused by autoimmune reactions. Expansion of beneficial bacterial families such as *Defluviitaleaceae* enhances antiviral immunity, it may prevent subacute thyroiditis and hypothyroidism by regulating the immune system (Zhang et al., 2024a). Concurrently, probiotics such as *Lactobacillus acidophilus* attenuate thyroid-specific autoantibody production (TPOAb and TgAb), curtailing autoimmune aggression against thyroid tissue (Weiss et al., 2010). This action reduces antigen translocation, and effectively blocks the source of systemic immune activation, thus providing a multifaceted strategy to mitigate immune-related pathologies.

Some probiotics can reduce the production of reactive oxygen species, enhance the activity of superoxide dismutase, glutathione peroxidase and other antioxidant enzymes (Ghoneim and Moselhy, 2016; Jin et al., 2024). Oxidative stress exacerbates thyroid cellular injury and fuels autoimmune activation. Probiotic supplementation significantly decreased serum oxidative stress markers while increasing antioxidant capacity in Hashimoto’s thyroiditis patients (da Silva et al., 2023). Enhanced oxidative resilience safeguards thyroid follicular cells, preserves hormone synthesis capacity, and supports long-term functional recovery in patients with hypothyroidism.

## 5 Challenges and future directions in microbiota-based interventions for hypothyroidism

**Figure 5:**
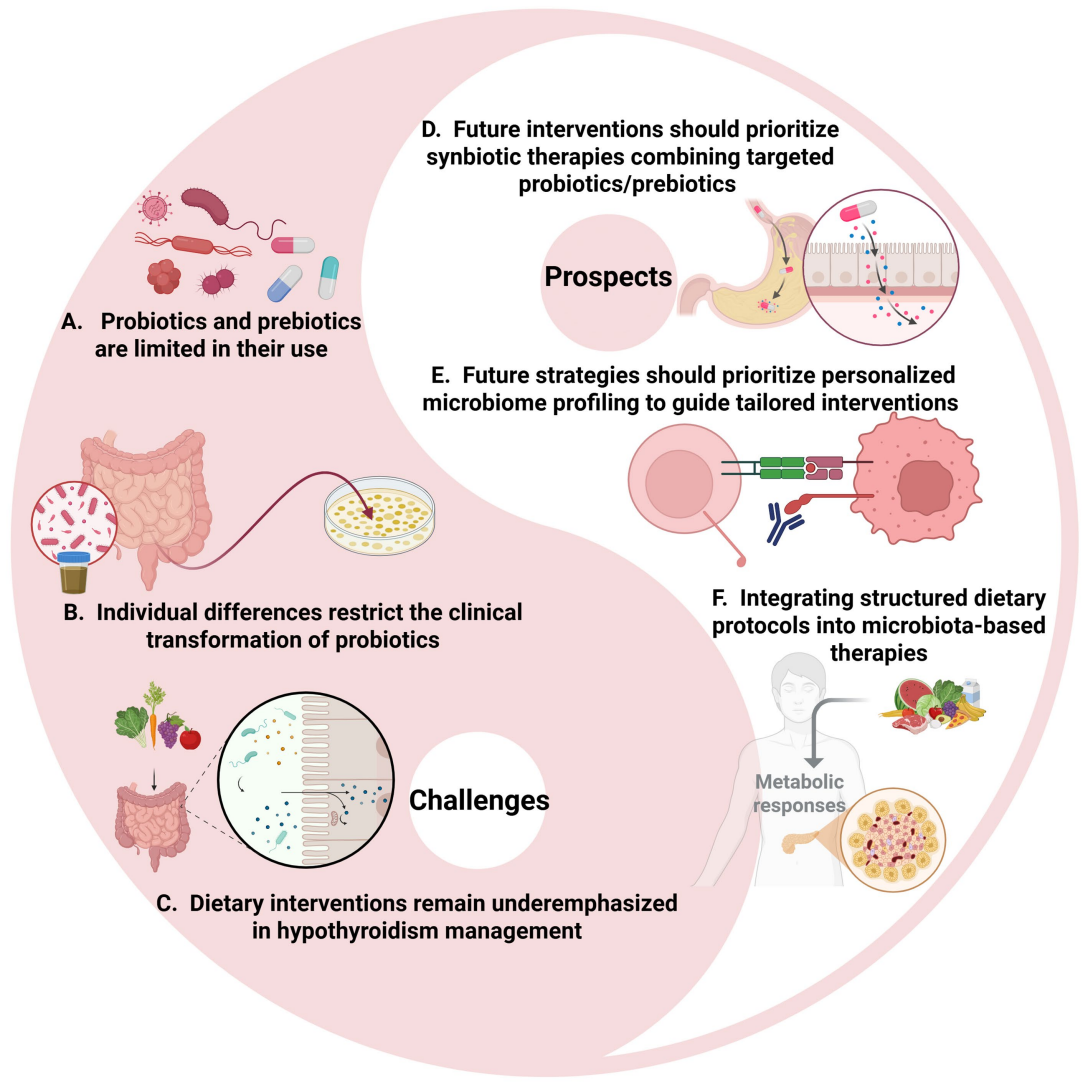
Challenges and prospects in hypothyroidism treatment. **(A–C)** Current challenges including the limited therapeutic application of probiotics and prebiotics, significant individual variability in clinical responses, and the lack of dietary intervention studies for hypothyroidism. **(D–F)** Future prospects including prompting our focus on exploring their unique benefits and developing personalized nutritional regimens to improve patients’ quality of life. Image source: used BioRender.com created.

Although microbiota-targeted strategies present a promising adjunct in the management of hypothyroidism, several critical challenges must be addressed to fully realize their therapeutic potential (Figure 5). These challenges reflect the inherent complexity of the gut-thyroid axis and the multifactorial nature of hypothyroidism pathogenesis.

### 5.1 Limited adoption of synbiotic and multi-modal approaches

Maintaining a healthy gut microbiota can help prevent and manage hypothyroidism, but existing intervention measures face certain challenges. Firstly, the application of probiotics and prebiotics is not widespread, only 20% of commercially available probiotic products contain prebiotic formulations (Sanders et al., 2019). While probiotics such as *Bifidobacterium* and *Lactobacillus* have shown potential in restoring gut microbial balance and modulating immune responses, their clinical application remains largely isolated, often lacking synergistic prebiotic supplementation (Ouyang et al., 2024). This fragmented approach limits the sustainability of therapeutic effects, particularly in hypothyroid patients with profound metabolic and inflammatory dysregulation.

To address this, future interventions should prioritize integrated synbiotic therapies, combining carefully selected probiotics with specific prebiotics to enhance microbial engraftment, stabilize gut ecology, and reinforce host metabolic and immune homeostasis (Balthazar et al., 2022). For example, for specific imbalances in the gut microbiota, corresponding probiotics should be selected for supplementation to restore the balance of the microbiota. Additionally, future studies should investigate the long-term effects of microbiome-based interventions and explore the potential of personalized medicine approaches tailored to individual patients’ gut microbial profiles (Cunningham et al., 2021).

### 5.2 The need for personalized microbiota-driven therapies

Significant interindividual variability in gut microbiota composition poses another challenge for standardized probiotic interventions. In hypothyroidism, particularly autoimmune forms, microbial alterations are dynamic and patient-specific. It is difficult to achieve personalized microbiome intervention, only 30% of clinical protocols are customized based on metagenomic data (Wu et al., 2023).

Future strategies should incorporate personalized microbiome profiling, enabling tailored interventions that target individual microbial deficiencies, optimize immune modulation, and maximize therapeutic efficacy (Porcari et al., 2023). These include the need to identify specific probiotic strains with the most significant therapeutic potential, determine optimal dosages and treatment durations, and establish standardized protocols for combining probiotics with thyroid medications. Based on the characteristics of the patient’s intestinal microbiome, personalized microbiome intervention plans should be formulated.

### 5.3 Underutilization of diet-based microbiota modulation

Despite substantial evidence linking diet to microbiota composition and function, dietary interventions remain underemphasized in hypothyroidism management. Diets rich in fiber, antioxidants, and anti-inflammatory nutrients, such as the Mediterranean diet, favor beneficial microbial communities, while high-fat, high-sugar diets exacerbate dysbiosis and inflammation (Vinelli et al., 2022; Farahbod et al., 2024). Dietary intervention has a significant impact on the composition of the gut microbiota.

Integrating structured dietary protocols into microbiota-based therapies offers a practical strategy to reinforce gut-thyroid axis stability, enhance intervention outcomes, and support long-term disease management. Currently, diets rich in antioxidants and anti-inflammatory components, such as the Mediterranean diet, are rarely used to improve the composition of the gut microbiota. Anti-inflammatory diets reduce the risk of autoimmune diseases, but the penetration rate is less than 15% (Khavandegar et al., 2024; Dimba et al., 2024).

## 6 Conclusion

The gut microbiota has emerged as a central player in thyroid health, exerting profound influence over immune regulation, nutrient absorption, and thyroid hormone metabolism. Dysbiosis-induced disruption of the gut-thyroid axis contributes significantly to the development and progression of hypothyroidism, particularly in autoimmune forms such as Hashimoto’s thyroiditis. Modulating the gut microbiota through targeted interventions, including probiotics, prebiotics, synbiotics, and dietary strategies, offers a promising avenue to restore microbial homeostasis, attenuate systemic inflammation, and improve the clinical management of hypothyroid disorders. While preliminary evidence supports the adjunctive use of microbiota-targeted therapies alongside conventional hormone replacement, challenges such as individualized intervention design, optimization of microbial formulations, and integration of dietary modulation must be addressed. Future efforts focused on mechanistic elucidation, precision microbiota profiling, and longitudinal clinical studies will be crucial for translating these emerging strategies into effective, personalized treatments for hypothyroidism.
